# Comprehensive Management of an Impacted Maxillary Central Incisor: A Case Report

**DOI:** 10.7759/cureus.71547

**Published:** 2024-10-15

**Authors:** Tanu Nangia, Gauri Kalra, Carrolene Langpoklakpam

**Affiliations:** 1 Pediatric Dentistry, Manav Rachna Dental College, School of Dental Sciences, Faridabad, IND

**Keywords:** central incisor, guided eruption, impaction, mixed dentition, supernumerary tooth

## Abstract

The impaction of a maxillary central incisor in young patients is rare but can significantly affect aesthetics, speech, and self-esteem. This case report details the diagnosis and treatment of an 11-year-old male patient with an impacted maxillary right central incisor, along with the presence of an impacted supernumerary tooth (mesiodens). The case was managed through a combination of surgical exposure of the impacted tooth and orthodontic traction to guide the impacted tooth into its correct position. Advances in diagnostic imaging and minimally invasive surgical techniques facilitated the successful resolution of the case. Early intervention in such cases is essential for preventing long-term functional and aesthetic complications.

## Introduction

The absence of a maxillary central incisor in a young patient is often a cause for aesthetic concern, both for the child and their parents. This missing tooth can have a profound impact on the child’s self-confidence and can lead to social discomfort. Parents and dental professionals frequently prefer to intervene early rather than waiting for the natural eruption of permanent teeth, as leaving the condition untreated can have long-term psychological and functional effects on the child.

Tooth impaction is a condition where a tooth does not emerge into the oral cavity within the expected time. The reasons for tooth impaction can vary widely, including both local and systemic factors. Impaction of maxillary central incisors is uncommon, despite the impaction of maxillary canines being more frequently seen in clinical settings. The incidence rate is low, with studies estimating that it occurs in only 0.06% to 0.2% of cases. The primary causes of such impaction are usually related to anatomical discrepancies or abnormalities [1]. Common factors contributing to impaction include retained deciduous teeth, the presence of supernumerary teeth, unusual eruption paths, and insufficient space in the dental arch [2].

The delayed eruption of a central incisor, although rare, can be diagnosed with higher accuracy during the mixed dentition period. The failure of an anterior tooth to erupt not only disrupts facial aesthetics but can also lead to difficulties in speech and function, as well as reduced self-esteem. Early intervention in these cases is often necessary to prevent further complications [3].

Recent advances in imaging and surgical techniques have significantly improved the management of impacted teeth. A study by Kolarkodi et al. (2023) [2] emphasizes the role of cone-beam computed tomography (CBCT) in identifying the precise location of impacted teeth and optimizing treatment plans [4]. This imaging modality provides a three-dimensional view, allowing clinicians to visualize the exact position of the impacted tooth, the surrounding bone, and any associated structures such as supernumerary teeth or cysts, which are frequently invisible on conventional radiographs. Additionally, minimally invasive surgical techniques, such as laser-assisted exposure, have been shown to reduce post-operative discomfort and improve periodontal outcomes in cases of impacted central incisors [4]. These advances underline the importance of incorporating new technologies into treatment plans to achieve more predictable results and minimize risks.

This particular case report discusses an 11-year-old boy who presented with a missing maxillary right central incisor. The situation was compounded by the presence of a supernumerary tooth in the vicinity of the impacted tooth. The case was managed through a comprehensive approach involving both surgical and orthodontic interventions to address the impaction and correct the positioning of the tooth. Such an interdisciplinary approach highlights the importance of early diagnosis and coordinated treatment in managing dental impactions effectively.

## Case presentation

**Figure 1:**
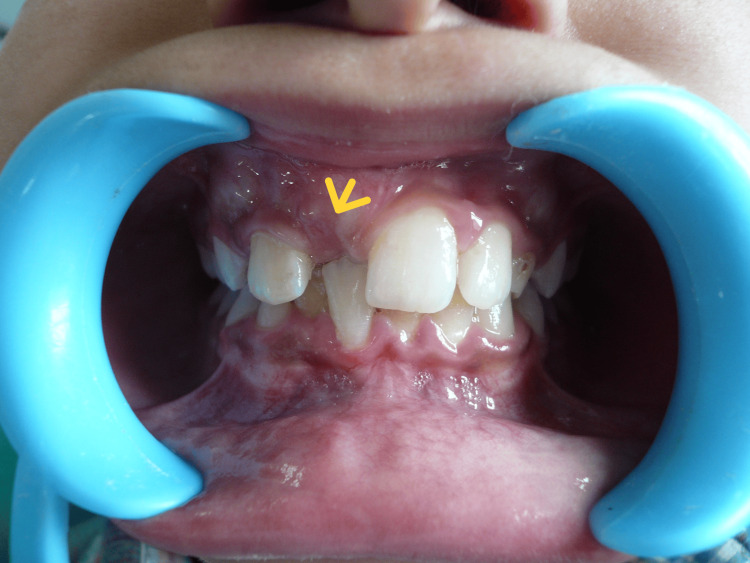
Pre-operative photograph Pre-operative image showing the missing maxillary right central incisor.

An 11-year-old male patient presented to the Department of Pediatric and Preventive Dentistry at Manav Rachna Dental College, Haryana, with a chief complaint of a missing front tooth (Figure 1).

**Figure 2:**
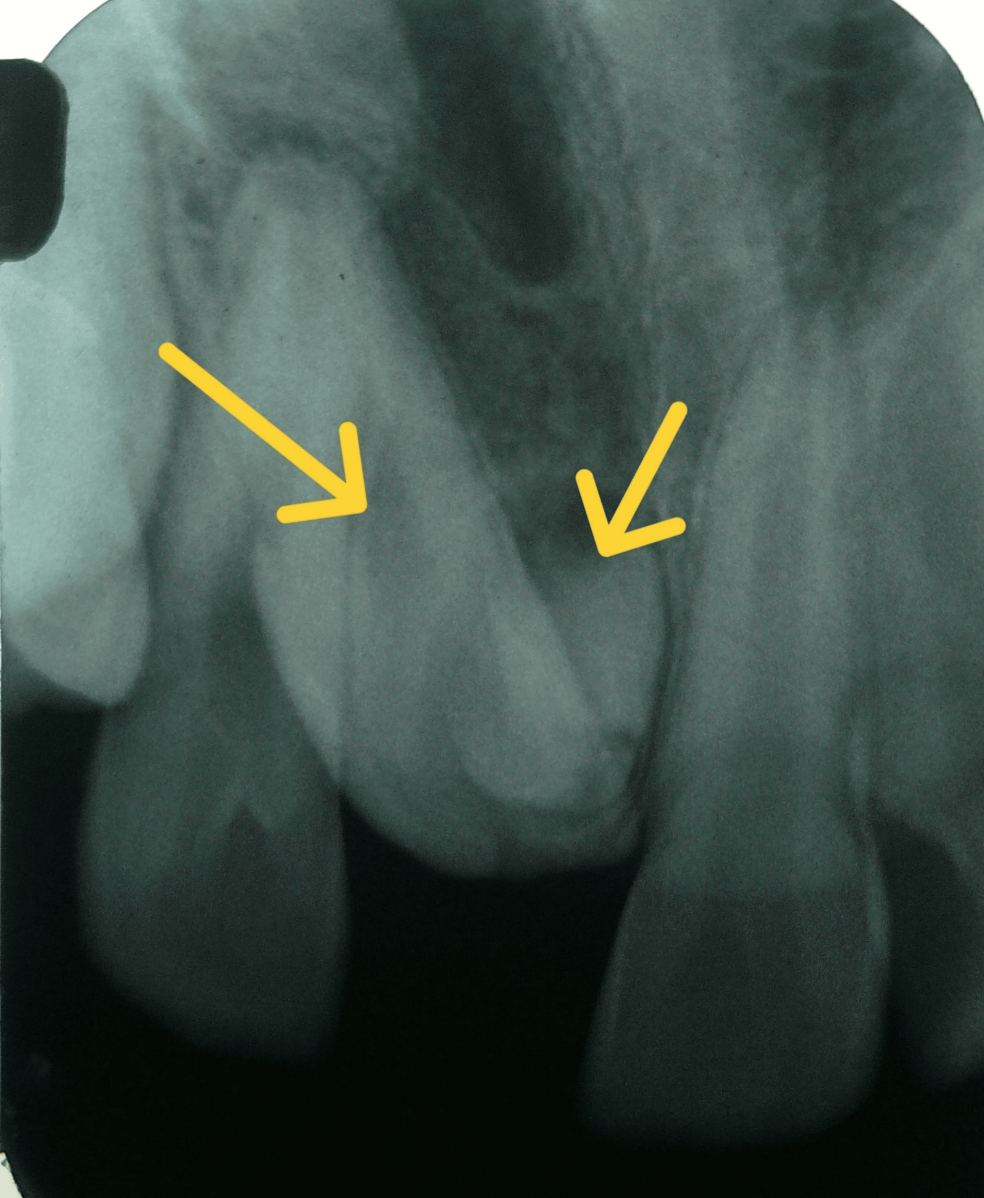
Intraoral periapical view Intra-oral periapical radiograph showing an unerupted maxillary right central incisor with an oblique path of eruption and a supernumerary bud lying adjacent to it.

**Figure 3:**
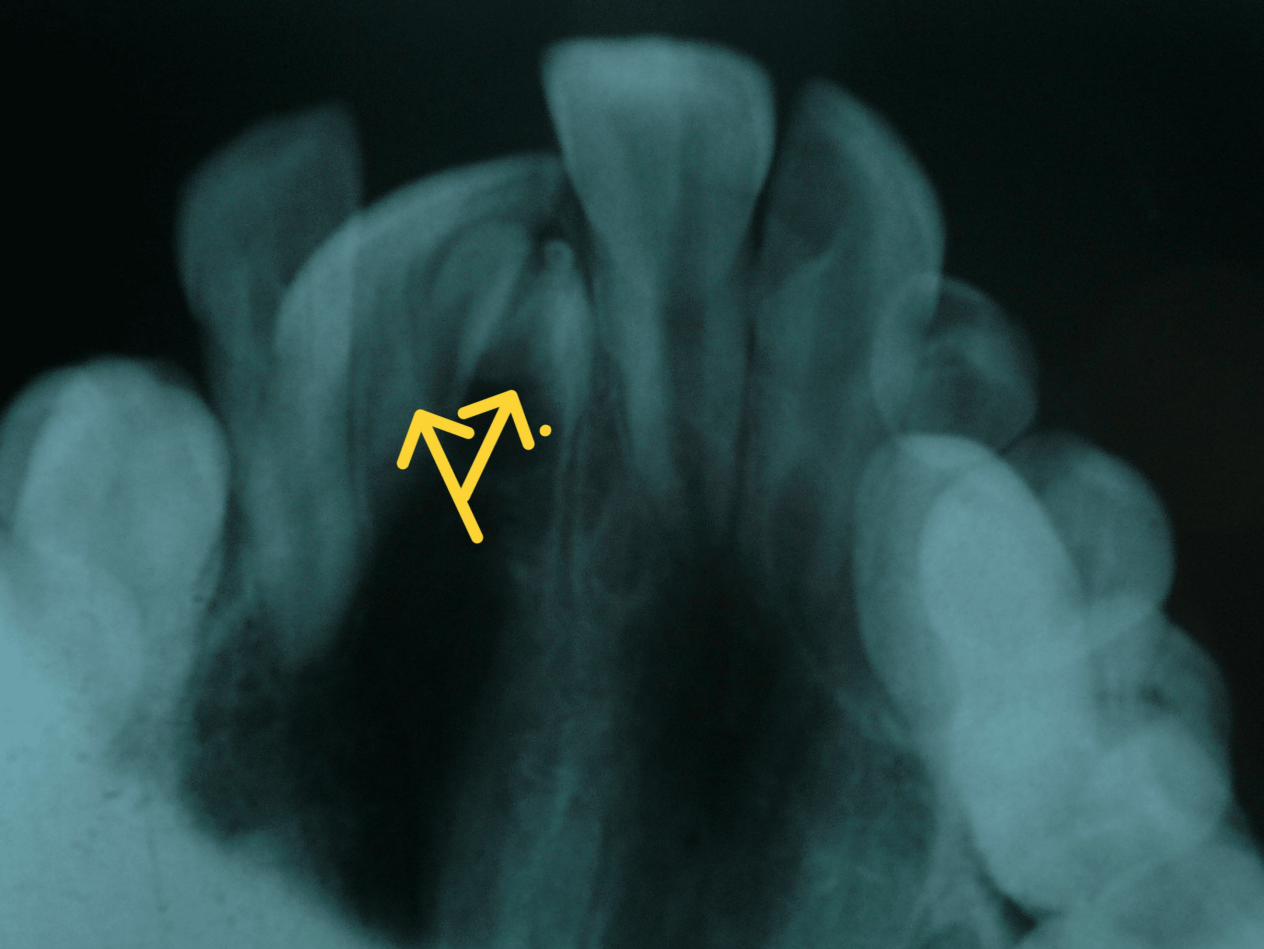
Maxillary occlusal view Maxillary occlusal radiograph showing impacted central incisor with supernumerary tooth bud. The supernumerary tooth was partially formed with no root formation seen.

The patient had no significant medical history and was otherwise in good health. Notably, the child had undergone a previous dental appointment a year back, however no change was observed. Upon clinical examination, the patient was found to be in the early mixed dentition phase. The maxillary right central incisor was absent, and there was no notable discrepancy in the arch length of either the maxilla or mandible. The patient exhibited a developing Angle’s class I molar relationship and a balanced facial profile, with adequate space for the impacted tooth to erupt. There was no evidence or history of any trauma or other complications. Radiographic assessment was carried out using an intraoral periapical radiograph (Figure 2) of the maxillary central incisor region and a maxillary occlusal view (Figure 3).

**Figure 4:**
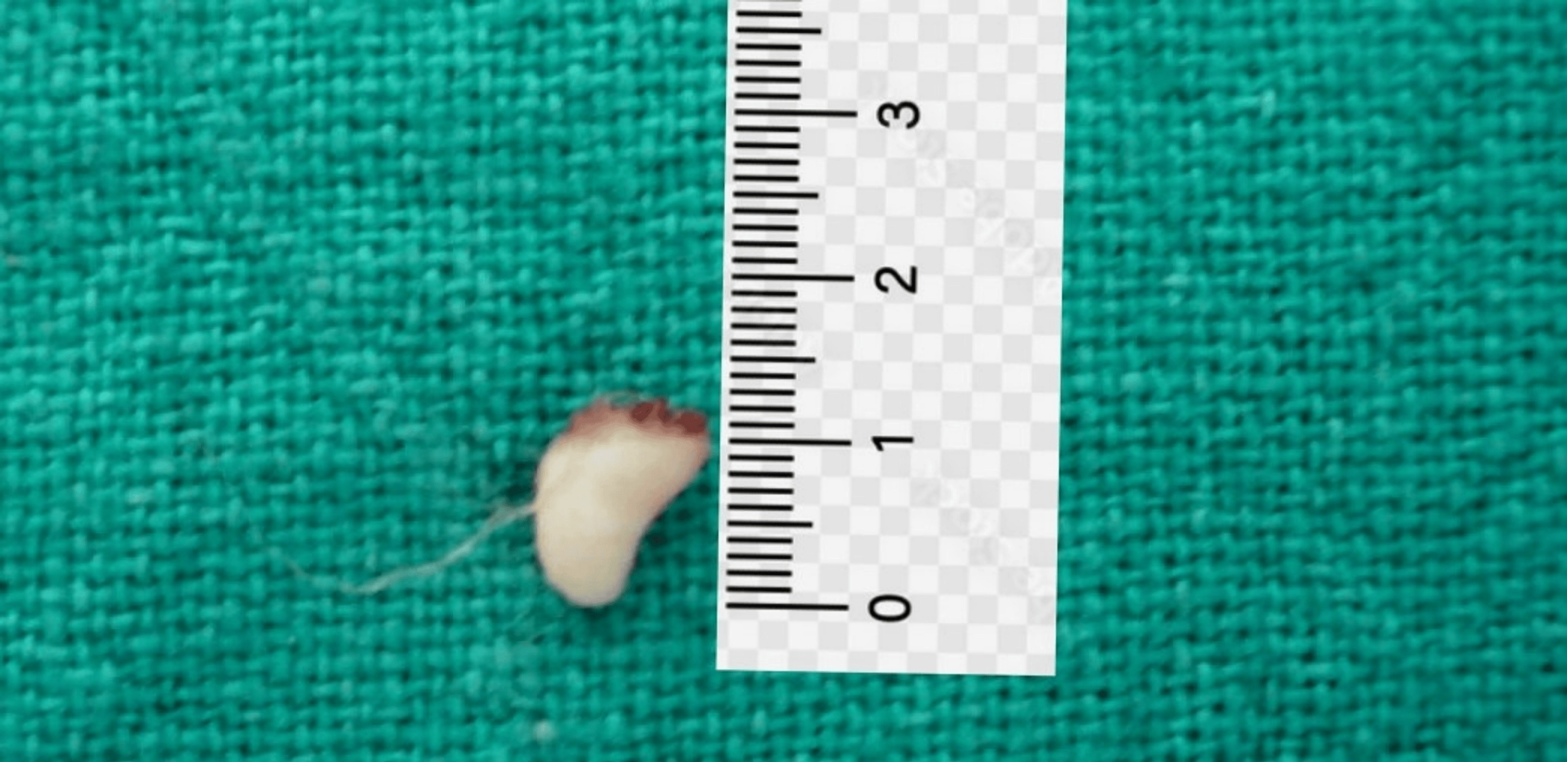
Extracted supernumerary tooth bud Extracted supernumerary tooth bud partially developed with no root formation and measuring around 10 mm

These images revealed an impacted maxillary right central incisor positioned obliquely, with the presence of a supernumerary tooth bud adjacent to its crown, obstructing the incisor's path of eruption. The impacted right permanent central incisor, however, was completely formed and no signs of ankylosis were visible on the radiograph. A supernumerary tooth obstructing the eruption of the permanent incisor was confirmed as one of the primary causes. An abnormal eruption path was also considered, as the tooth's trajectory had been altered by the adjacent supernumerary tooth. Written and informed consent was obtained from the parents. The treatment plan initially involved the surgical removal of the supernumerary tooth and the exposure of the impacted central incisor. A full-thickness mucoperiosteal flap was raised, and the supernumerary tooth was extracted without complications (Figure 4).

A gingival window was created over the crown of the impacted incisor in the same appointment using a No. 11 BP blade to expose the tooth. An edgewise orthodontic bracket was bonded to the impacted incisor through the window created to allow for orthodontic traction without requiring additional surgical intervention. Bleeding was arrested, sutures were placed, and the patient was recalled after seven days for suture removal.

**Figure 5:**
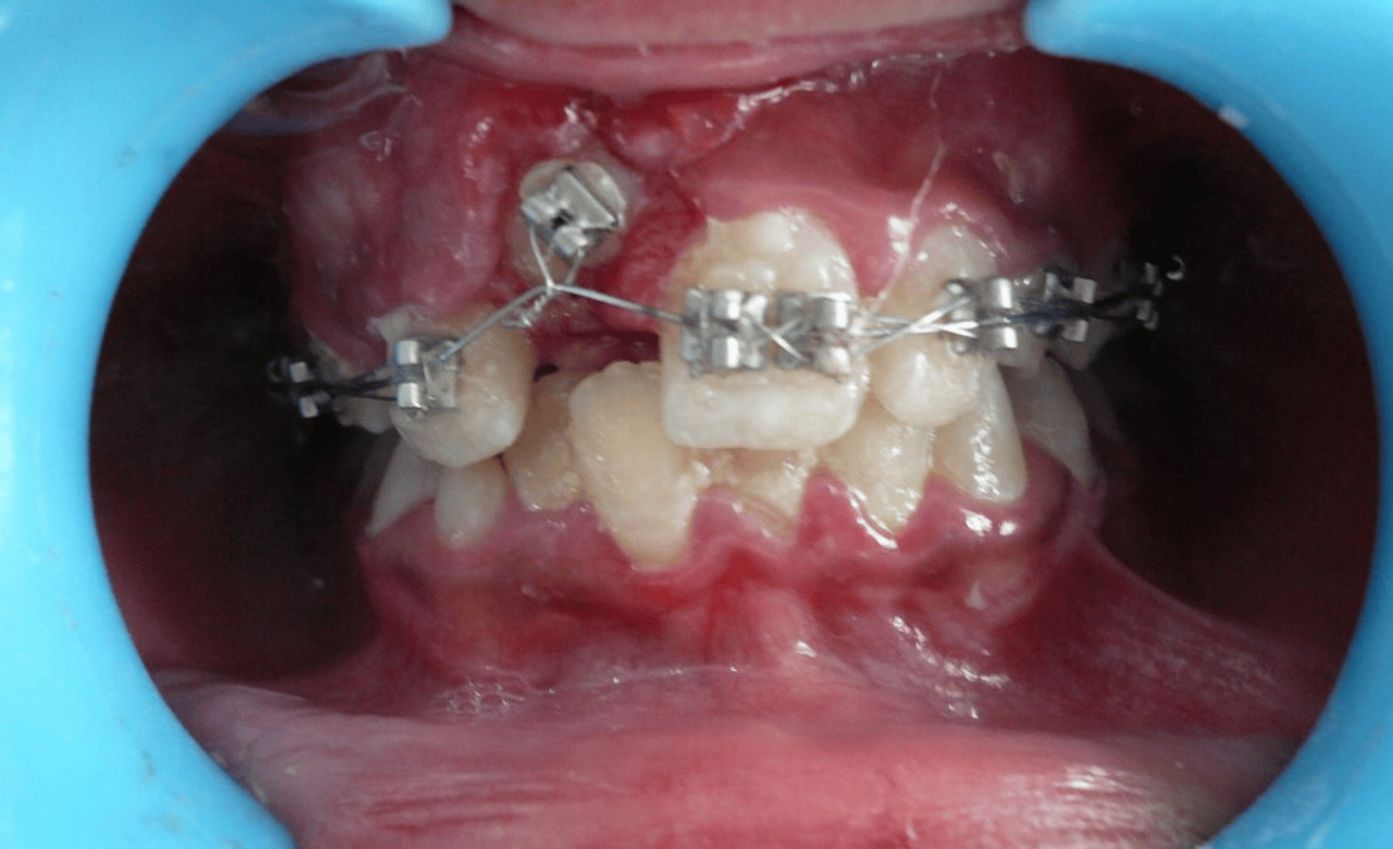
Intra-operative photograph - orthodontic repositioning Initial positioning of brackets and archwire to initiate orthodontic repositioning

The tooth was initially left to erupt spontaneously, and the patient was scheduled for regular follow-up visits at weekly intervals. Despite regular monitoring, no significant movement of the impacted tooth was observed after four weeks. Given the patient’s dental history, it was decided to initiate orthodontic repositioning. Molar bands were adapted on the upper first molars, and pre-adjusted edgewise brackets were bonded to all the teeth (Figure 5).

**Figure 6:**
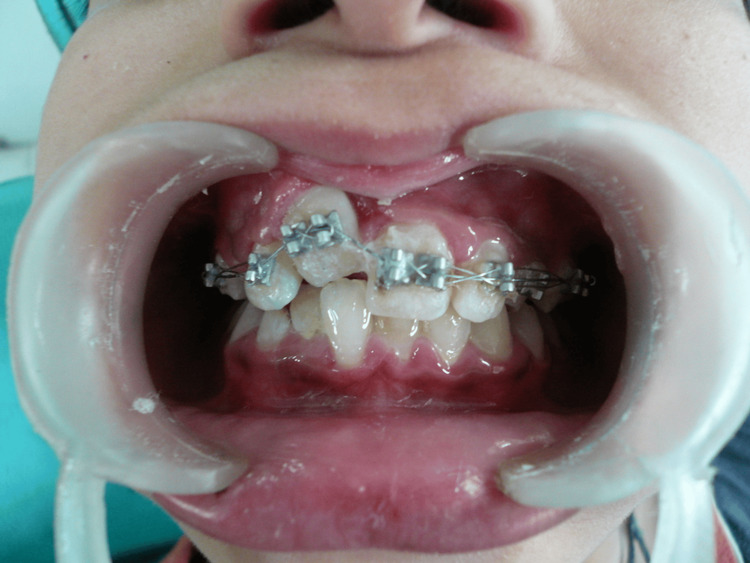
Two weeks follow up Two weeks follow-up showing movement of impacted central incisor in the oral cavity

An initial 0.014-inch archwire was engaged in all the brackets, and the impacted tooth was ligated to the main archwire to begin its movement. The patient was recalled every two weeks for adjustments. Within two weeks, the impacted tooth began to show signs of movement (Figure 6).

**Figure 7:**
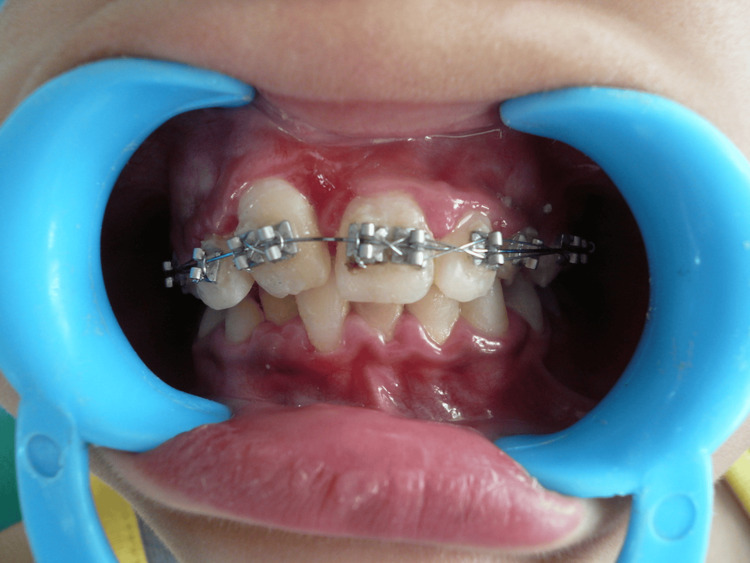
Repositioning of the tooth in the arch Follow-up showing repositioning of the tooth in the oral cavity

At each follow-up appointment, the ligature was tightened, and within six weeks, the tooth had fully erupted to its occlusal level. Minor orthodontic adjustments were made using a 0.016-inch archwire and a 0.018 x 0.025 rectangular wire to ensure proper alignment and stability (Figure 7).

**Figure 8:**
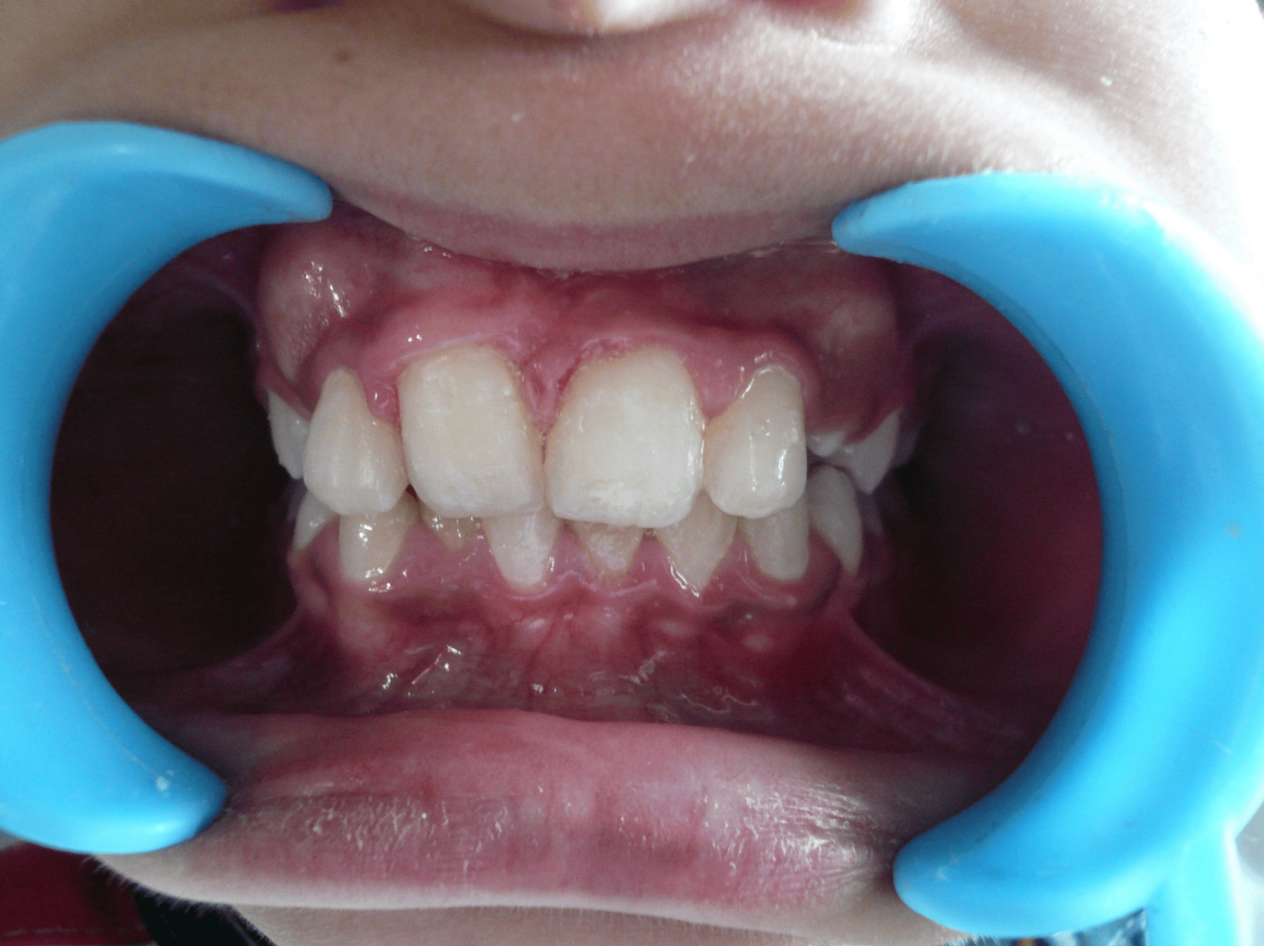
Post-operative view Post-operative image at 16 weeks showing fully erupted right central incisor in a functional position.

The treatment was completed successfully within 16 weeks from the date of surgical intervention performed, with the tooth in a functional and aesthetically acceptable position (Figure 8).

## Discussion

Although the impaction of maxillary central incisors is less frequent than maxillary canines, it can cause considerable concern during the mixed dentition phase due to the lack of anterior tooth eruption. This delay negatively impacts facial aesthetics and can lower a child’s self-esteem, speech development, and social interactions. Local factors such as arch length discrepancies, supernumerary teeth, and bony or mucosal barriers have been identified as common causes of tooth impaction [4]. Supernumerary teeth, in particular, are found to be the primary cause of upper incisor impaction, contributing to approximately 56-60% of cases [5,6]. These extra teeth often physically obstruct the normal eruption path, leading to the displacement of neighbouring teeth and malformations in root development [7].

According to Seehra et al. (2023) [8], spontaneous eruption can occur over up to three years, though the need for adjunctive orthodontic intervention is common to achieve proper alignment within the arch. The current literature emphasizes the importance of early intervention in cases of impacted central incisors, as it significantly reduces the overall treatment time and complexity.

In certain cases, removing a supernumerary tooth alone may not be sufficient to ensure the eruption of the impacted incisor. This necessitates further surgical intervention to expose the impacted tooth, followed by orthodontic traction to guide the tooth into its correct position [9]. While effective, repeated surgical interventions can affect periodontal health, resulting in gingival defects or contour irregularities post-treatment [7]. Hence, it is important to assess the periodontal condition when planning such procedures carefully.

Several treatment options are available for managing impacted maxillary incisors, including extraction followed by restorative procedures such as implants or bridges, orthodontic space closure, or surgical exposure with orthodontic guidance [9]. The choice of treatment is dependent on factors like the patient’s age, the position of the impacted tooth, and overall dental development. Early diagnosis and timely intervention are critical for minimizing treatment complexity and duration [10].

In this case, the combination of surgical exposure and orthodontic traction successfully resolved the impaction. The approach achieved acceptable outcomes in terms of aesthetics, occlusion, and periodontal health. Consistent with the study by Alqahtani et al. (2021) [11], this method demonstrated that early intervention offers significant advantages in terms of periodontal health preservation and overall treatment efficiency. Continuous monitoring post-treatment is essential to ensure stability and evaluate periodontal health over the long term [12].

The timely and successful management of the impacted maxillary central incisor in this 11-year-old patient illustrates the importance of early diagnosis and interdisciplinary treatment. By addressing the impaction through surgical exposure, removal of the obstructing supernumerary tooth, and orthodontic traction, we not only corrected the dental anomaly but also helped prevent potential long-term consequences. Clinically, this treatment restored the child’s facial aesthetics, significantly improving their self-esteem and social confidence during a critical developmental stage.

Furthermore, the functional benefits of early intervention were clear. Correcting the tooth position at this stage ensured proper dental arch alignment, preserving speech clarity and masticatory function. This approach minimized the risk of future complications such as malocclusion, periodontal disease, or abnormal wear of adjacent teeth, which could have occurred if the impacted tooth had been left untreated.

In this case, early intervention also reduced the treatment time and complexity, as delaying treatment might have led to more invasive procedures or the need for prosthetic replacements. The use of orthodontic traction in conjunction with surgical exposure allowed for a less aggressive approach, preserving the periodontal health of the impacted tooth and contributing to its successful eruption. Regular follow-up and adjustments were critical in ensuring the tooth’s gradual and stable movement into its correct position.

Overall, this case demonstrates that addressing impacted teeth early in life not only resolves immediate aesthetic and functional concerns but also plays a crucial role in the child's psychosocial development, ensuring a healthier and more confident smile for years to come.

## Conclusions

Early diagnosis and interdisciplinary management of the impacted maxillary central incisor in this case restored both function and aesthetics, boosting the child’s confidence. Timely intervention helped prevent potential speech, occlusal, and periodontal complications. The combination of surgical exposure and orthodontic traction ensured a successful outcome with minimal long-term risks. This approach highlights the importance of early treatment in preserving overall oral health.
